# A TALE of shrimps: Genome-wide survey of homeobox genes in 120 species from diverse crustacean taxa

**DOI:** 10.12688/f1000research.13636.1

**Published:** 2018-01-17

**Authors:** Wai Hoong Chang, Alvina G. Lai

**Affiliations:** 1 Nuffield Department of Medicine, University of Oxford, Oxford, OX3 7FZ, UK

**Keywords:** Crustacean, homeobox, TALE, comparative genomics, arthropod, homeodomain

## Abstract

The homeodomain-containing proteins are an important group of transcription factors found in most eukaryotes including animals, plants and fungi. Homeobox genes are responsible for a wide range of critical developmental and physiological processes, ranging from embryonic development, innate immune homeostasis to whole-body regeneration. With continued fascination on this key class of proteins by developmental and evolutionary biologists, multiple efforts have thus far focused on the identification and characterization of homeobox orthologs from key model organisms in attempts to infer their evolutionary origin and how this underpins the evolution of complex body plans. Despite their importance, the genetic complement of homeobox genes has yet been described in one of the most valuable groups of animals representing economically important food crops. With crustacean aquaculture being a growing industry worldwide, it is clear that systematic and cross-species identification of crustacean homeobox orthologs is necessary in order to harness this genetic circuitry for the improvement of aquaculture sustainability. Using publicly available transcriptome data sets, we identified a total of 4183 putative homeobox genes from 120 crustacean species that include food crop species, such as lobsters, shrimps, crayfish and crabs. Additionally, we identified 717 homeobox orthologs from 6 other non-crustacean arthropods, which include the scorpion, deer tick, mosquitoes and centipede. This high confidence set of homeobox genes will now serve as a key resource to the broader community for future functional and comparative genomics studies.

## Introduction

As one of the fastest growing industries, the seafood trade is dominated by fishing and farming of crustaceans, with annual sales exceeding $40 billion ( Stentiford *et al*., 2012). Crustacean aquaculture is multi-faceted, not only contributing to the ever-increasing demands by international markets, but is also directly linked to the socio-economic aspects of many developing nations through the creation of jobs and infrastructure. Aquaculture practices have intensified in recent years to cope with the demand. Yet, many are not sustainable since the increased densities of farmed shrimps often serve as hotbeds for pathogens if left unabated, causing infectious diseases and the devastation of cultures resulting in massive financial losses. As a result, regulations associated with aquaculture diseases are being enforced with emphasis placed on preventative measures, e.g. enhancement of broodstock and research aiming to further our understanding on crustacean development and ways to utilize the innate ability of crustaceans to combat pathogens ( Lai & Aboobaker, 2017; Stentiford *et al*., 2012).

Several conserved molecular genetic circuitries are well-known for regulating many aspects of development and innate immune homeostasis. One prominent example would be homeobox genes, a family of transcription factors defined by the presence of a homeodomain ( Holland, 2013). As one of the most important master controls in development, some headway has already been made in understanding the involvement of homeobox genes in innate immunity; Caudal in *Drosophila melanogaster* is implicated in commensal-gut mutualism ( Ryu *et al*., 2004; Ryu *et al*., 2008). Given their importance, major efforts have thus far focused on characterization of homeobox genes in well-known model organisms such as humans ( Garcia-Fernàndez, 2005; Holland *et al*., 2007), *Caenorhabditis elegans* ( Bürglin, 1997), *D. melanogaster* ( Mukherjee & Bürglin, 2007), planarians ( Currie *et al*., 2016; Felix & Aboobaker, 2010; Garcia-Fernandez *et al*., 1991), amphioxus ( Luke *et al*., 2003), teleost fish ( Mulley *et al*., 2006) and many more. Although homeobox orthologs have been previously studied in the crustacean *Parhyale hawaiensis* ( Kao *et al*., 2016), systematic and cross-species characterization of this gene family across the broader Crustacea with focus on food crop species is currently lacking. A better understanding of homeobox genes in crustaceans is therefore required to address this major shortfall, leading us to our present work.

## Methods

### Transcriptome data sets and query sets

We retrieved complete transcriptome data sets for 120 crustacean species available at the time of manuscript preparation from the European Nucleotide Archive. Six non-crustacean arthropod proteomes were retrieved from Uniprot. A complete list of accessions used in this study is provided in Supplementary Table 1. We retrieved a list of query sequences used in subsequent homology searches from Uniprot and GenBank.

### Identification of homeobox orthologs

Based on a previously published workflow ( Lai & Aboobaker, 2017), we used multiple Basic Local Alignment Search Tool ( BLAST)-based approaches, such as BLASTp and tBLASTn to identify genes with homeodomain sequences. The BLAST results were filtered by e-value of < 10 ^-6^, best reciprocal BLAST hits against the GenBank non-redundant (nr) database and redundant contigs having at least 95% identity were collapsed using CD-HIT. We then utilized HMMER (version 3.1) employing hidden Markov models (HMM) profiles ( Finn *et al*., 2011) to scan for the presence of Pfam homeodomains ( Bateman *et al*., 2004) on the best reciprocal nr BLAST hits, to compile a final non-redundant set of crustacean and arthropod homeobox gene orthologs ( Dataset 1).

### Multiple sequence alignment and phylogenetic tree construction

Multiple sequence alignment of homeodomain sequences was performed using MAFFT (version 7) ( Katoh *et al*., 2009). Phylogenetic tree was built from the MAFFT alignment using RAxML WAG + G model to generate a best-scoring maximum likelihood tree ( Stamatakis, 2014). Geneious (version 7) was used to generate a graphical representation of Newick tree ( Kearse *et al*., 2012).

## Results and discussion

### Identification of putative homeobox genes in crustaceans

**Figure 1.**
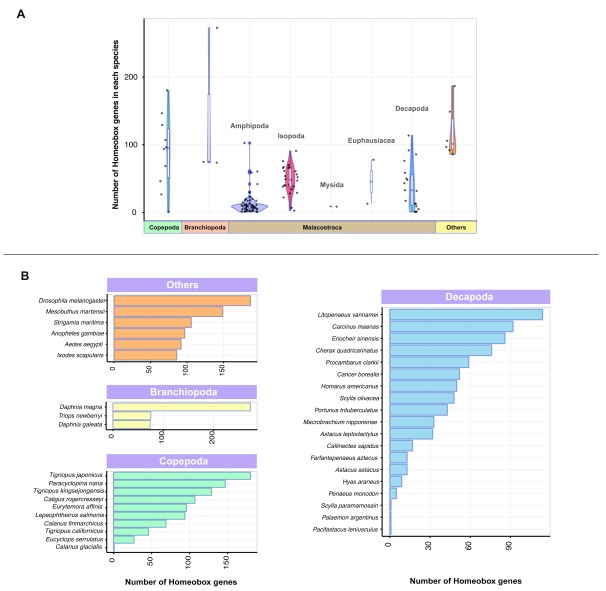
The homeobox superfamily in Crustacea and representative arthropod species. ( **A**) Number of homeobox gene orthologs identified in each species are depicted as boxplots, indicating the median and quartiles. Violin plots underlying the boxplots illustrate sample distribution across different crustacean taxa and kernel probability density (width of the shaded areas represent the proportion of data located in these areas). The homeobox gene orthologs from six non-crustacean species within Arthropoda (others) are also shown. ( **B**) Bar charts illustrating the number of homeobox gene orthologs in crustaceans from Decapoda, Branchiopoda and Copepoda along with six non-crustacean arthropods (others).

With the recent availability of a large number of transcriptome data sets, we perform an extensive search for homeobox genes from 120 crustacean species. We focus on species represented across the broader Crustacea sampling from three main crustacean classes, Malacostraca, Branchiopoda and Copepoda, with focus on key food crop species from the order Decapoda ( Supplementary Table 1). Using BLAST-based approaches and profile HMM ( Bateman *et al*., 2004; Finn *et al*., 2011; Finn *et al*., 2015) for homology searches, we conservatively identified 4183 transcripts with homeodomain sequences from crustaceans ( Figure 1; Dataset 1). Additionally, we included six non-crustacean arthropod species in our search and from these species, we identified 717 homeobox orthologs ( Figure 1; Dataset 1).

List of Pfam annotated homeobox genes and associated e-values in crustaceans and other arthropods Click here for additional data file. Copyright: © 2018 Chang WH and Lai AG 2018 Data associated with the article are available under the terms of the Creative Commons Zero "No rights reserved" data waiver (CC0 1.0 Public domain dedication).

Fasta file for homeobox gene sequences in crustaceans and other arthropods Click here for additional data file. Copyright: © 2018 Chang WH and Lai AG 2018 Data associated with the article are available under the terms of the Creative Commons Zero "No rights reserved" data waiver (CC0 1.0 Public domain dedication).

### Classification and phylogenetic analysis of TALE class genes

**Figure 2.**
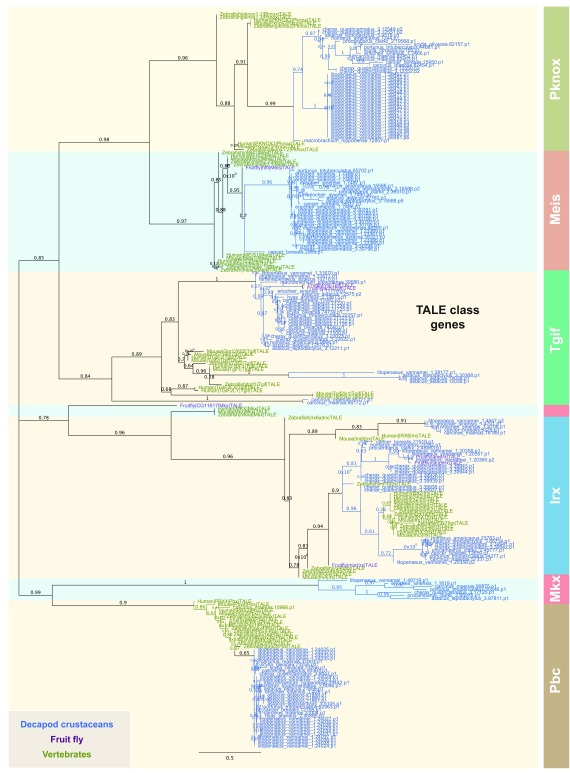
Phylogeny of TALE superclass orthologs in decapod crustaceans. The tree was constructed using the maximum-likelihood method from an amino acid multiple sequence alignment, which include TALE class genes from other species ( Zhong *et al*., 2008 and Zhong & Holland, 2011). TALE orthologs representing 6 subclasses are colour-coded. The node labels of each taxon are marked with distinctive colors denoted in the figure inset. Bootstrap support values (n=1000) are denoted as branch labels.

Concerted efforts to establish evolutionary classification of homeobox genes have resulted in 11 recognised classes ( Edvardsen *et al*., 2005; Holland *et al*., 2007; Ryan *et al*., 2006; Zhong *et al*., 2008; Zhong & Holland, 2011). The Three-Amino acid-Loop Extension (TALE) superclass within the group of homeobox genes is characterized by three additional residues between alpha helices 1 and 2 of the homeodomain ( Bertolino *et al*., 1995). TALE class homeodomain proteins are further divided into 6 subclasses, Meis, Pknox, Pbc, Irx, Mkx and Tgif characterized by distinct motifs beyond the homeodomain ( Bürglin, 1997; Bürglin, 2005; Holland *et al*., 2007; Mukherjee & Bürglin, 2007). We have classified a total of 165 TALE class orthologs from 15 decapod crustacean species ( Figure 2). These genes form distinct phylogenetic grouping, which allows confident assignment of decapod TALE class orthologs into 6 sub-families ( Figure 2). Importantly, the tree topology of crustacean TALE class orthologs recapitulated observations from a previous study ( Holland *et al*., 2007).

## Conclusion

We identified 4900 homeodomain transcripts from 120 crustaceans and 6 non-crustacean arthropod species. Although this data set is non-exhaustive – transcriptomes contain only genes expressed at the point of sample collection – it will now serve as a key resource for future functional studies in the context of crustacean aquaculture. Beyond crustaceans, this work is widely applicable to studies on homeobox genes from other animals and will facilitate evolutionary and comparative genomics investigations. 

## Data availability

The data referenced by this article are under copyright with the following copyright statement: Copyright: © 2018 Chang WH and Lai AG

Data associated with the article are available under the terms of the Creative Commons Zero "No rights reserved" data waiver (CC0 1.0 Public domain dedication). 

Dataset 1: List of Pfam annotated homeobox genes and associated e-values in crustaceans and other arthropods. DOI, 10.5256/f1000research.13636.d190417 ( Chang & Lai, 2018).

Dataset 2: Fasta file for homeobox gene sequences in crustaceans and other arthropods. DOI, 10.5256/f1000research.13636.d190418 ( Chang & Lai, 2018).

## Supplementary material

**Supplementary Table 1:** List of accession numbers for species used in this study.

## References

[ref-1] Bateman A Coin L Durbin R: The Pfam protein families database. 2004;32(Database issue):D138–41. 10.1093/nar/gkh121 14681378 PMC308855

[ref-2] Bertolino E Reimund B Wildt-Perinic D: A novel homeobox protein which recognizes a TGT core and functionally interferes with a retinoid-responsive motif. 1995;270(52):31178–31188. 10.1074/jbc.270.52.31178 8537382

[ref-3] Bürglin TR: Analysis of TALE superclass homeobox genes (MEIS, PBC, KNOX, Iroquois, TGIF) reveals a novel domain conserved between plants and animals. 1997;25(21):4173–4180. 10.1093/nar/25.21.4173 9336443 PMC147054

[ref-4] Bürglin TR: Homeodomain proteins. 2005.

[ref-5] Chang WH Lai AG: Dataset 1 in: A TALE of shrimps: Genome-wide survey of homeobox genes in 120 species from diverse crustacean taxa. 2018 Data Source 10.12688/f1000research.13636.1 PMC5968366 29899973

[ref-6] Chang WH Lai AG: Dataset 2 in: A TALE of shrimps: Genome-wide survey of homeobox genes in 120 species from diverse crustacean taxa. 2018 Data Source 10.12688/f1000research.13636.1 PMC5968366 29899973

[ref-7] Currie KW Brown DD Zhu S: HOX gene complement and expression in the planarian *Schmidtea mediterranea*. 2016;7:7. 10.1186/s13227-016-0044-8 27034770 PMC4815179

[ref-8] Edvardsen RB Seo HC Jensen MF: Remodelling of the homeobox gene complement in the tunicate *Oikopleura dioica* . 2005;15(1):R12–R13. 10.1016/j.cub.2004.12.010 15649342

[ref-9] Felix DA Aboobaker AA: The TALE class homeobox gene Smed-prep defines the anterior compartment for head regeneration. 2010;6(4):e1000915. 10.1371/journal.pgen.1000915 20422023 PMC2858555

[ref-10] Finn RD Clements J Arndt W: HMMER web server: 2015 update. 2015;43(W1):W30–W38. 10.1093/nar/gkv397 25943547 PMC4489315

[ref-11] Finn RD Clements J Eddy SR: HMMER web server: interactive sequence similarity searching. 2011;39(Web Server issue):W29–37. 10.1093/nar/gkr367 21593126 PMC3125773

[ref-12] Garcia-Fernàndez J: The genesis and evolution of homeobox gene clusters. 2005;6(12):881–892. 10.1038/nrg1723 16341069

[ref-13] Garcia-Fernandez J Baguñà J Saló E: Planarian homeobox genes: cloning, sequence analysis, and expression. 1991;88(16):7338–7342. 10.1073/pnas.88.16.7338 1714599 PMC52290

[ref-14] Holland PW: Evolution of homeobox genes. 2013;2(1):31–45. 10.1002/wdev.78 23799629

[ref-15] Holland PW Booth HA Bruford EA: Classification and nomenclature of all human homeobox genes. 2007;5:47. 10.1186/1741-7007-5-47 17963489 PMC2211742

[ref-16] Kao D Lai AG Stamataki E: The genome of the crustacean *Parhyale hawaiensis*, a model for animal development, regeneration, immunity and lignocellulose digestion. 2016;5: pii: e20062. 10.7554/eLife.20062 27849518 PMC5111886

[ref-17] Katoh K Asimenos G Toh H: Multiple alignment of DNA sequences with MAFFT. 2009;537:39–64. 10.1007/978-1-59745-251-9_3 19378139

[ref-18] Kearse M Moir R Wilson A: Geneious Basic: an integrated and extendable desktop software platform for the organization and analysis of sequence data. 2012;28(12):1647–1649. 10.1093/bioinformatics/bts199 22543367 PMC3371832

[ref-19] Lai AG Aboobaker AA: Comparative genomic analysis of innate immunity reveals novel and conserved components in crustacean food crop species. 2017;18(1):389. 10.1186/s12864-017-3769-4 28521727 PMC5437397

[ref-20] Luke GN Castro LF McLay K: Dispersal of NK homeobox gene clusters in amphioxus and humans. 2003;100(9):5292–5295. 10.1073/pnas.0836141100 12704239 PMC154338

[ref-21] Mukherjee K Bürglin TR: Comprehensive analysis of animal TALE homeobox genes: new conserved motifs and cases of accelerated evolution. 2007;65(2):137–153. 10.1007/s00239-006-0023-0 17665086

[ref-22] Mulley JF Chiu CH Holland PW: Breakup of a homeobox cluster after genome duplication in teleosts. 2006;103(27):10369–10372. 10.1073/pnas.0600341103 16801555 PMC1502464

[ref-23] Ryan JF Burton PM Mazza ME: The cnidarian-bilaterian ancestor possessed at least 56 homeoboxes: evidence from the starlet sea anemone, Nematostella vectensis. 2006;7(7):R64. 10.1186/gb-2006-7-7-R64 16867185 PMC1779571

[ref-24] Ryu JH Kim SH Lee HY: Innate immune homeostasis by the homeobox gene *caudal* and commensal-gut mutualism in *Drosophila*. 2008;319(5864):777–782. 10.1126/science.1149357 18218863

[ref-25] Ryu JH Nam KB Oh CT: The homeobox gene *Caudal* regulates constitutive local expression of antimicrobial peptide genes in *Drosophila* epithelia. 2004;24(1):172–185. 10.1128/MCB.24.1.172-185.2004 14673153 PMC303351

[ref-26] Stamatakis A: RAxML version 8: a tool for phylogenetic analysis and post-analysis of large phylogenies. 2014;30(9):1312–1313. 10.1093/bioinformatics/btu033 24451623 PMC3998144

[ref-27] Stentiford GD Neil DM Peeler EJ: Disease will limit future food supply from the global crustacean fishery and aquaculture sectors. 2012;110(2):141–157. 10.1016/j.jip.2012.03.013 22434002

[ref-28] Zhong YF Butts T Holland PW: HomeoDB: a database of homeobox gene diversity. 2008;10(5):516–518. 10.1111/j.1525-142X.2008.00266.x 18803769

[ref-29] Zhong YF Holland PW: HomeoDB2: functional expansion of a comparative homeobox gene database for evolutionary developmental biology. 2011;13(6):567–568. 10.1111/j.1525-142X.2011.00513.x 23016940 PMC3399086

