# Supplementary material for: A TALE of shrimps: Genome-wide survey of homeobox genes in 120 species from diverse crustacean taxa
Source: F1000Res. 2018 Jan 17;7:71. [Version 1] doi: 10.12688/f1000research.13636.1 (PMC5968366; doi:10.12688/f1000research.13636.1)
Supplement: Peer review history

# Peer review: Referee response for version 1

**DOI:** 10.5256/f1000research.14814.r33514

**Published:** 2018-05-23

**Authors:** Colleen Doherty

**Affiliations:** 1 Department of Molecular and Structural Biochemistry, North Carolina State University, Raleigh, NC, USA

In this manuscript, Chang and Lai identify sequences of the homeobox genes in crustaceans from transcriptional data. For TALE family members, they classify these orthologs into the six subfamilies. The introduction provides the justification for establishing this resource in these agriculturally important species. In total, this will be an important reference source for future work in understanding the transcriptional regulation of development in crustacean species.  There are a few minor questions and suggestions the authors can address.

 The details of the BLAST-based approaches and CD-Hit should be described in more detail. Were default settings applied? Perhaps adding an additional supplemental file or link with the commands would clarify this to facilitate replication in other species.  

 The authors have the opportunity to address how effective their approach is and potential places for improvement. For example, how do the datasets derived from proteomes compare to that from transcriptomes?  Is there an overlapping dataset where the two approaches could be compared?  Also, how does the identification of homeobox genes using this approach in Drosophila compare to the number annotated in this well-studied species?  If all are identified this would suggest that this approach may be sufficient if some are missing it suggests that this conservative starting point could be enhanced in the future by additional refinement.  

 Minor comments:

 Introduction, second paragraph, description of homeobox identification in other species. Either provide references for “and many more” or omit.  As is, this phrase is vague and doesn’t add constructively to the introduction.

I have read this submission. I believe that I have an appropriate level of expertise to confirm that it is of an acceptable scientific standard.

# Peer review: Referee response for version 1

**DOI:** 10.5256/f1000research.14814.r32836

**Published:** 2018-04-13

**Authors:** Nathan J Kenny

**Affiliations:** 1 Department of Life Sciences, The Natural History Museum of London, Cromwell Road, London, SW7 5BD, UK

This work makes a putative assessment of the overall homeodomain complements of the transcriptomes of a number of crustacean species. One class of homeodomain containing genes (TALE) from one order of crustaceans (Decapoda) is assessed in detail, but otherwise no attempt is made to categorise putative hits into gene families. The work is therefore preliminary in scope, and would benefit from the provision of even broadest-level classification of hits into appropriate classes/subclasses of gene, which should be fairly straightforward given the diagnostic residues used to categorise these classes.

 The title as it stands is misleading - a genome wide survey is not made, and instead transcriptomic data is used, which will (by necessity) be gappy.

 A link is made between aquaculture, innate immunity and homeodomain-containing proteins, but this is very tenuous. Particularly, why the focus is on TALE class genes is unclear, if *Caudal* is the gene used as the exemplar for a link between these fields?

 The methods section needs to be more precise. For example: "such as BLASTp and tBLASTn to identify genes with homeodomain sequences". What was done? How were protein sequences derived from nucleotide data for blastp searches? What sequences were used to search your datasets? (Perhaps add these to the sentence:  "list of query sequences used in subsequent homology searches from Uniprot and GenBank."). The latter is particularly important as more distant sequences may be missed.

 I have several questions about e values.

 -Dataset 1 contains several ID'd genes with higher E values than the stated cutoff (< 10-6). Is this deliberate?

 -The e-value of < 10-6 will also likely result in larger datasets returning more hits, purely as a consequence of how the E (expect) value is calculated. For example, the *Daphnia magna *transcriptome has 271,000 sequences, *Triops* 12,000. Therefore it is much more likely that sequences will make it through your annotation pathway in *Daphnia magna* rather than *Triops*. This will skew the results shown in Fig 1B. Is it possible to show that homeodomain genes are not artificially excluded, perhaps by giving the *E* values and best blast hits from "next-best" excluded sequences in your initial searches of small datasets, to prove no homeodomain sequences were artificially excluded? This is crucial, given the short length of the homeodomain, which will be the primary source of signal.

 Was HMMR really run on the best reciprocal nr hits, as is suggested by your phrasing?  Or was it run on the transcriptome-derived data?

 Fig 1A: Violin plots are not appropriate here. Look for instance at the Branchiopod data, where 3 points are used to infer this plot.

 The results in the tree in Fig 2 seem to indicate that decapod crustaceans completely lack Mkx genes, and the presentation of this is disingenuous in text (note the paraphyly of known Mkx homologues with regard to the inferred crustacean Mkx). Instead, the crustacean Mkx seem to be Pbc?

 "Importantly,  the  tree  topology  of  crustacean  TALE class  orthologs  recapitulated  observations  from  a  previous  study (Holland et al., 2007)." - this statement does not seem to be correct.

 The homeodomain complements, especially of ANTP class genes, of several crustacean species have been described previously, but no attempt is made to place the results observed in the context of the annotated sets of other species. Could this be provided? Particularly, the utility of the re-assessment of non-crustacean datasets is unclear, as these resources have been annotated previously in more detail. Were additional homeodomain-containing genes found by this re-analysis? Or fewer?

 In short, this work seems to be partially successful in its aims. With the addition of additional information about the identity of sequences, and the correction of the problems noted above, it will be a coherent addition to extant information on crustacean homeodomain-containing genes.

***Minor comments:***

  $40 billion - which currency? USD?

 There are several areas where the phrasing could be improved, e.g.

 -"With continued fascination on this key class of proteins"

 Sometimes articles (a/the) are missing from the text, e.g.

 - "Phylogenetic tree was built from"

I have read this submission. I believe that I have an appropriate level of expertise to confirm that it is of an acceptable scientific standard, however I have significant reservations, as outlined above.

# Peer review: Referee response for version 1

**DOI:** 10.5256/f1000research.14814.r29917

**Published:** 2018-02-14

**Authors:** Ricardo M. Zayas

**Affiliations:** 1 Department of Biology, San Diego State University (SDSU), San Diego, CA, USA

The report by Chang and Lai provide an extensive dataset and phylogenetic analysis crustacean homeobox genes. This data will be useful to individuals interested in studying the evolution and function of homeobox genes in crustacea and other organisms. Overall, this manuscript is well written and appears to be technically sound. I only have some very minor comments the authors could address to improve clarity:

 1) In the abstract, the rationale for the work takes an intellectual leap: it is unclear how identification of homeobox genes will be useful for aquaculture sustainability. I think the authors provide some compelling reasons within the text of the manuscript.

 2) In the results and discussion there are a few instances where the authors should use the past tense.

 3) Page 3, last sentence: even though the authors referred to Peter Holland's work, they should be much more precise about the observations from a "previous study". The authors should expand what they mean.

I have read this submission. I believe that I have an appropriate level of expertise to confirm that it is of an acceptable scientific standard.
